# Retroperitoneal paraganglioma with metastasis to the abdominal vertebra: a case report

**DOI:** 10.1186/1746-1596-8-52

**Published:** 2013-03-28

**Authors:** Jun He, Xingjun Wang, Wei Zheng, Yaowang Zhao

**Affiliations:** 1 Department of Urology, Hunan Children Hospital, Changsha 41007, China; 2 Department of Urology, Second Xiangya Hospital, Central South University, Changsha, Hunan 410011, China; 3 Hunan Children Hospital, Changsha 410007, China

**Keywords:** Paraganglioma, Retroperitoneum, Metastasis, Vertebra

## Abstract

**Background:**

Extra-adrenal paraganglioma of the retroperitoneum with metastasis to the vertebra is very rare. To our knowledge this is the first report of this kind of disease in the literature.

**Case presentation:**

Here, we present an oroginal case of paraganglioma of the retroperitoneum with metastasis to the abdominal vertebra in a 42-year-old female patient who was successfully treated by complete removal of the tumor and its metastasis. The patient was followed up for four years and remained disease-free.

**Conclusion:**

Our case demonstrated the need to consider paraganglioma of the retroperitoneum in the differential diagnosis of retroperitoneal mass, metastatic tumors to the vertebra, and the importance of radical surgery for a successful management of the disease.

**Virtual Slides:**

The virtual slide(s) for this article can be found here: http://www.diagnosticpathology.diagnomx.eu/vs/1956611954880197

## Case report

Paragangliomas, also known as extra-adrenal pheochromocytomas, are rare neuroendocrine neoplasms that are derived from the paraganglia, a diffuse neuroendocrine system dispersed from the skull base to the pelvic floor. They are found in many tissues such as the adrenal medulla, the carotid bodies, the organs of Zuckerkandl, and the paraganglia of the sympathetic and parasympathetic neurons [1,2]. Paragangliomas usually occur in the head and neck region but rare in the retroperitoneum. It has been estimated that as much as 10% paragangliomas of the retroperitoneum arise outside of the adrenal gland. In addition, few cases regarding paragangliomas of the retroperitoneum with metastases have been reported [3].

**Figure 1:**
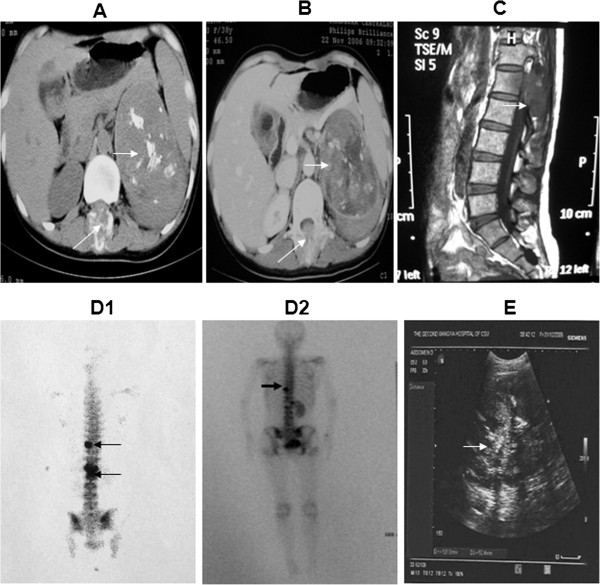
**CT scan (A) and contrast enhanced CT scan (B) showed an inhomogeneous density of mass (10.5×6.7 cm^2^) in the left adrenal region, with cystic changes, scattered calcified foci (arrow) and inhomogeneous enhancement (arrow).** The mass is ventral to the left kidney and the abdominal aorta. (**C**) MRI showed a well-defined mass and showed a mass in the back of vertebra (arrow). (**D1**) SPECT showed Tc-99m-MDP highly accumulated in the tenth of thoracic vertebra and the first and second lumbar (arrow). (**D2**) SPECT showed that the whole body bone scintigraphy is clear, with no abnormal structure detected. On the left half of the ninth thoracic a radionuclide uptake dot is visible. Left renal agenesis is seen (arrow). (**E**) Ultrasonographic study further demonstrates the mass has an uneven internal echo (arrow). The mass is irregular in shape and without a distinct boundary, between the left kidney and spleen.

Here, a rare case of paraganglioma of the retroperitoneum with metastasis to the abdominal vertebra was reported in a 42-year-old female patient. The patient had an increasing degree of pain in left lower back for over a month with a blood pressure of 106/60 mmHg. An immobile, hard but smooth mass below the left costal margin was diagnosed by physical examination. This solidmass was palpated in the first lumbar vertebra without a distinct boundary. Computed Tomography (CT) scan of the mass showed an inhomogeneous density (10.5×6.7 cm^2^) in the left adrenal region with cystic changes. The scattered spots indicated the calcification foci. No lymph node swelling and distant metastasis were detected except for the lesion in the first lumble vertebra. Osteolytic bone destruction and punctiform dead bone was detected in the first lumbar vertebra, which affect the posterior margin of the vertebra, bilateral arch and plate, superior and inferior articular processus, processus spinosus and left processus transverses (Figure 1A and 1B). Magnetic Resonance Imaging (MRI) of the first lumbar vertebra showed a well-defined mass. The mass was isointense on T1 and hypointense on T2 (Figure 1C). Furthermore, Single Photon Emission Computed Tomography (SPECT) detected that Technetium-99m methylene diophosphate (Tc-99m-MDP) highly accumulated in the tenth of thoracic vertebra and the first and second lumbar vertebra (Figure 1D1). Moreover, ultrasonography displayed an irregular, boundary-less and uneven internal echo mass between left kidney and spleen (Figure 1E). Vanillyl mandelic acid (VMA) and catecholamine in the urine and blood of the patient was in the normal range. The preoperative diagnosis was an adrenal tumor with metastasis to the abdominal vertebra.

**Figure 2:**
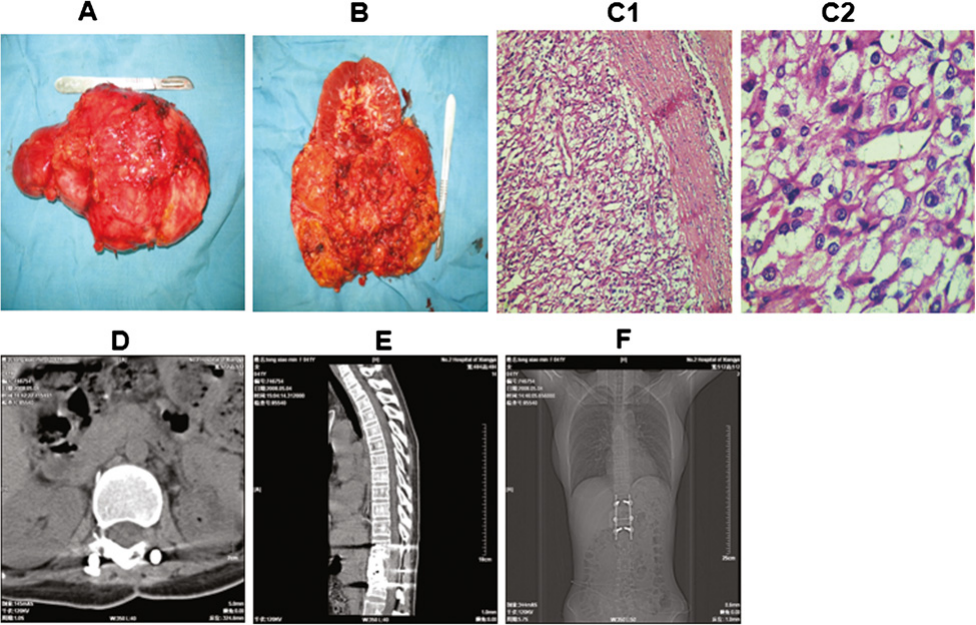
**Gross appearance of the resected tumor and the left kidney.** The tumor is about 10×9×7cm^3^, appeared solid, and is well vascularized. Macroscopically, no apparent tumor invasion into the kidney was observeded (**A**, **B**). The resected tumor was stained with H&E method. The tumor cells contained fine eosinophilic granules, mitotic figures and some nuclear atypia. **C1** (× 100). **C2** (× 400). Resection of the lumbar tumor and treated by posterior fixation with Moss Miami system (**D**, **E**, **F**).

**Figure 3:**
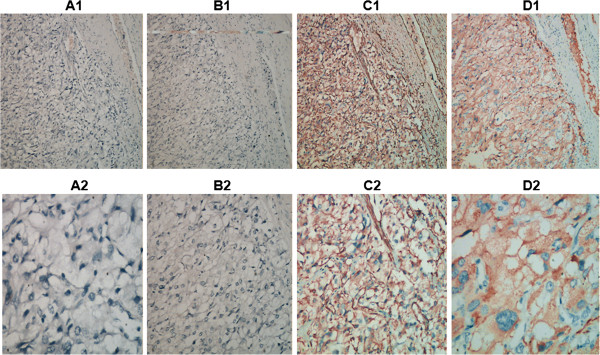
**Neither cell type was positive for cytokeratin or EMA (A1, × 100; A2, × 400; B1, ×100; B2, × 400).** Cell Cytoplasm showed positive reactivity for vimentin and ChromograninA (**C1**, × 100; **C2**,× 400; **D1**, × 100; **D2**, × 400).

**Figure 4:**
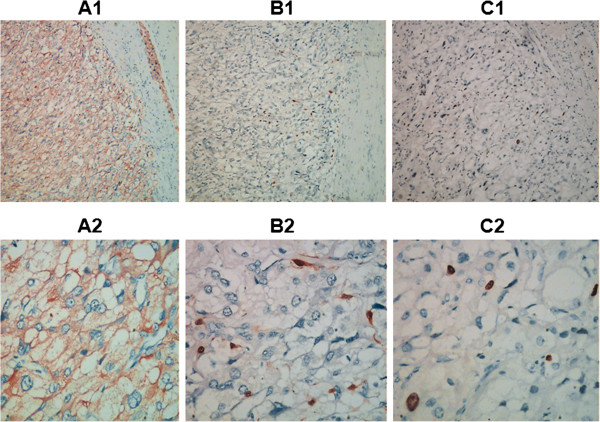
**Cell cytoplasm showed positive reactivity for synaptophysin (A1, ×100; A2, × 400).** Individual support cells showed positive reactivity for S-100 protein **(B1**, × 100; **B2**, × 400). Ki-67 labeling index was less than 1% (**C1**, × 100; **C2**, × 400).

The primary lesion was resected through a thoracoabdominal incision in December 2006. Intraoperative examination showed that the stomach, transverse colon, pancreas and spleen were displaced by the mass, but without significant adhesion to the ventral surface of the tumor. The left kidney tightly adhered to the tumor and was difficult to separate from it. The volume of the solid tumor is about 10×9×7 cm^3^ with integrated surface and full of vascular network. The left kidney and the tumor were resected and the weight of the tumor is 670 grams. Intraoperative blood loss is 300 ml. The intersection of the tumor is red-brown, full of blood vessels, fibrous septa and bone-like substance with some schistic hemorrhage and necrosis in the middle. Macroscopically, no invasion of the tumor into the kidney can be observed. Histologically, the tumor was alveolar-like with many well-vascularized septa (Figure 2A and 2B). The tumor cells contained fine eosinophilic granules, mitotic figures and some nuclear atypia (Figure 2C1, Figure 2C2). Immunohistochemical examination of the tumor was as follows: cytokeratin- (Figure 3A1, 3A2), epithelial membrane antigen (EMA)- (Figure 3B1, 3B2), vimentin+ (Figure 3C1, 3C2), S-100 sported+ (Figure 3D1, 3D2), synaptophysin+ (Figure 4A1, 4A2), chromograninA+ (Figure 4B1, 4B2),The Ki-67 labeling index was less than 1%.(Figure 4C1, 4C2),These findings led to a histological diagnosis of non-chromaffin paraganglioma of the retroperitoneum.

After two weeks, the patient was transferred to the Department of Spine Surgery for resection of the lumbar tumor and treated by posterior fixation with Moss Miami system (Figure 2D, 2E, 2F). Intraoperative blood loss is 3000 ml. Pathological examination showed metastasis of the paraganglioma to the vertebra. The patient has been followed up for four years and does not show any sign of recurrence (Figure 1D2).

## Discussion

The clinical, pathologic and radiological features of retroperitoneal paragangliomas have been previously described [4,5]. Paraganglioma can be diagnosed in early stage if it is a functional tumor secreting excessive amounts of catecholamine. For those patients with a nonfunctional paraganglioma, VMA and catecholamine are normal in her urine and blood. Diagnosis of retroperitoneal paraganglioma could be delayed as it usually relies on the growth of the tumor mass. It is hard to make a correct preoperative diagnosis due to absence of typical clinical symptoms in the patient.

CT, MRI or ultrasonographic studies are sensitive in detecting a retroperitoneal mass and could delineate its location, outline, internal structure as well as its relationship with the surrounding organs. However, specific diagnosis for the retroperitoneal mass still relies on postoperative histopathological diagnosis. In addition, histopathological diagnosis is required to define the paraganglioma as benign or malignant tumours exhibit similar clinical diagnosis and imaging findings [6]. Chromogranin A and synaptophysin are the most common neuropeptides synthesized in endocrine cells and can be used for immunohistochemical analysis of paragangliomas along with other protein markers such as neuron specific enolase and vimentin. They can aid the correct diagnosis of this rare disease. Our immunohistochemical analysis revealed that the tumor was positive for Chromogranin A, vimentin, S-100, synaptophysin, thus providing a histopathological basis for a correct diagnosis of nonchromaffin paraganglioma of the retroperitoneum in the patient. Zhao et al. described a case of pigmented paraganglioma showing weak expression of chromogranin A, but the tumor cells were negative for vimentin and devoid of the S-100 protein positive cells [7]. Okubo et al. report a case of duodenal gangliocytic paraganglioma tumor cells showing positive response for synaptophysin [8]. In these studies [7,8], the Ki-67 labeling index was less than 1%, which is similar to our results.

Most paragangliomas are benign in nature and the malignancy rate is about 10%to 15% [9]. The presence of metastatic lesions is considered to be the only acceptable fact for malignant paraganglioma and is used to discriminate between malignant and benign paraganglioma. The combination of a large size, heterogeneous density, and irregular margins has been suggested to have an increased likelihood of malignancy [4]. In addition, an extra-adrenal tumor location and high tumor weight (more than 80 grams) were also considered to have an increased chance of malignant paraganglioma [10]. Metastasis of paraganglioma of the retroperitoneum is usually to lungs, lymph nodes, liver, bones, or the spleen., The presence of paraganglia tissue in these organs strongly indicates a metastasis. We are aware of one reported case regarding paraganglioma of the retroperitoneum with direct invasion of the spinal canal which lead a cross section syndrome of the cauda equine [11]. It has been no previous report of metastasis to the lumbar vertebra on paraganglioma of the retroperitoneum. Our case suggests that metastatic paraganglioma of the retroperitoneum should be considered as a rare case of metastases in the differential diagnosis of vertebral masses.

Management paragangliomas of the retroperitoneum with metastases remains a very challenging task as complete surgical removal of the tumor as well as the metastasis offer the only chance for cure and no other therapeutic methods have been shown to provide long-term survival. Meticulous and complete surgical removal of the original tumor and its metastasis has been reported to associate with long-term survival [12]. Most paragangliomas have an intact capsule with abundant blood vessels both on its surface and inside. Therefore, most paragangliomas can be removed without much difficulty if the tumor is meticulously resected. A thoraco-abdominal incision is indicated if an adrenal tumor is large [13], which provides an adequate exposure of the tumor mass in the peritoneal cavity and greater maneuverability for operation. The patient does not have any sign of tumor recurrence after four years of surgery, suggesting the surgery is successful and effective. Our results demonstrate the need to consider paraganglioma of the retroperitoneum in the differential diagnosis of retroperitoneal mass and in the differential diagnosis of metastatic tumors to the vertebra. They also illustrate the importance of radical surgery for a successful management of paraganglioma of the retroperitoneum with metastasis.

## Consent

Written informed consent was obtained from the patient for publication of this Case Report and any accompanying images. A copy of the written consent is available for review by the Editor-in Chief of this journal.

## Competing interests

The authors declare that they have no competing interests.

## Authors’ contributions

JH and X-JW carried out pathological examination and wrote the manuscript as major contributors. WZ participated in pathological investigations. Y-WZ carried out the immunohistochemical staining and collected the patient’s clinical information. JH gave final approval to the manuscript as a corresponding author. All authors have read and approved the final manuscript.
